# Tuberculosis and HIV are the leading causes of adult death in northwest Ethiopia: evidence from verbal autopsy data of Dabat health and demographic surveillance system, 2007–2013

**DOI:** 10.1186/s12963-017-0139-z

**Published:** 2017-07-17

**Authors:** Yigzaw Kebede, Gashaw Andargie, Abebaw Gebeyehu, Tadesse Awoke, Mezgebu Yitayal, Solomon Mekonnen, Mamo Wubshet, Temesgen Azmeraw, Yihunie Lakew, Kassahun Alemu

**Affiliations:** 1 Department of Epidemiology and Biostatistics, Institute of Public Health, University of Gondar, PoBox, 196, Gondar, Ethiopia; 2 Department of Health Service Management, Institute of Public Health, University of Gondar, Gondar, Ethiopia; 3 Department of Reproductive Health, Institute of Public Health, University of Gondar, Gondar, Ethiopia; 4 Department of Human Nutrition, Institute of Public Health, University of Gondar, Gondar, Ethiopia; 5 Department of Environmental and Occupational Health and Safety, Institute of Public Health, University of Gondar, Gondar, Ethiopia; 6 Dabat Health and Demographic Surveillance Site, Institute of Public Health, University of Gondar, Gondar, Ethiopia; 7 Ethiopian Public Health Association, Addis Ababa, Ethiopia

**Keywords:** Adult, Dabat, Death, HDSS, Verbal autopsy, Ethiopia

## Abstract

**Background:**

Reliable data on causes of death form the basis for building evidence on health policy, planning, monitoring, and evaluation. In Ethiopia, the majority of deaths occur at home and civil registration systems are not yet functional. The main objective of verbal autopsy (VA) is to describe the causes of death at the community or population level where civil registration and death certification systems are weak and where most people die at home without having had contact with the health system.

**Methods:**

Causes of death were classified and prepared based on the International Classification of Diseases (ICD-10). The cause of a death was ascertained based on an interview with next of kin or other caregivers using a standardized questionnaire that draws information on signs, symptoms, medical history, and circumstances preceding death. The cause of death, or the sequence of causes that led to death, is assigned based on the data collected by the questionnaire. The complete VA questionnaires were given to two blinded physicians and reviewed independently. A third physician was assigned to review the case when disagreements in diagnosis arose.

**Results:**

Communicable diseases (519 deaths [48.0%]), non-communicable diseases (377 deaths [34.8%]), and external causes (113 deaths [10.4%]) were the main causes of death between 2007 and 2013. Of communicable diseases, tuberculosis (207 deaths [19.7%]), HIV/AIDS (96 deaths [8.9%]) and meningitis (76 deaths [7.0%]) were the most common causes of death.

**Conclusion:**

Tuberculosis, HIV/AIDS, and meningitis were the most common causes of deaths among adults. Death due to non-communicable diseases showed an increasing trend. Increasing community awareness of infections and their interrelationships, tuberculosis case finding, effective local TB programs, successful treatment, and interventions for HIV are supremely important.

## Background

Data on causes of death are important for mapping the geographical burden of disease and for health sector planning, including assessing programmatic needs, monitoring progress of interventions, and reassessing health priorities. Cause of death data are crucial for understanding the overall epidemiological profile of disease in a population [1, 2].

It is reported that more than two-thirds of deaths in the world occur in developing countries but causes of death are not well documented, and vital registration systems are nonexistent or at rudimentary stages [3, 4]. Many deaths occur at home, outside the formal health sector, and few are attended by qualified medical professionals. Verbal autopsy (VA) has been used to assign causes of death in such settings [4]. In recent years, concern has been raised about the inadequate attention to public health interventions and investment to prevent some health problems in adults [2, 5]. Different studies found changing trends in all-cause and disease-specific mortality among adults in sub-Saharan African (SSA) countries [6–8]. While most global efforts to prevent mortality among young people focus on children under 5 years of age, significant health gains can also be attained among adults. However, targeted efforts for adults are hindered by a lack of data [5, 9].

Ethiopia is one of the SSA countries with no functional vital registration system yet. Additionally, health service utilization is very poor, with total outpatient use of government health facilities estimated at 0.3 visits per person per year [8]. Thus, due to poor access to health services and low health-care-seeking behavior, most deaths occur outside of health facilities. As a result, mortality data in communities are lacking.

Cognizant of the lack of information on basic demographic and health outcomes, the Dabat Health and Demographic Surveillance System (HDSS) was established, with the purpose of, among other things, knowing community-level causes of death. This study therefore aimed to assess the causes of adult deaths in the population covered by the Dabat HDSS using VA data between 2007 and 2013.

## Methods

### Study setting

**Fig. 1:**
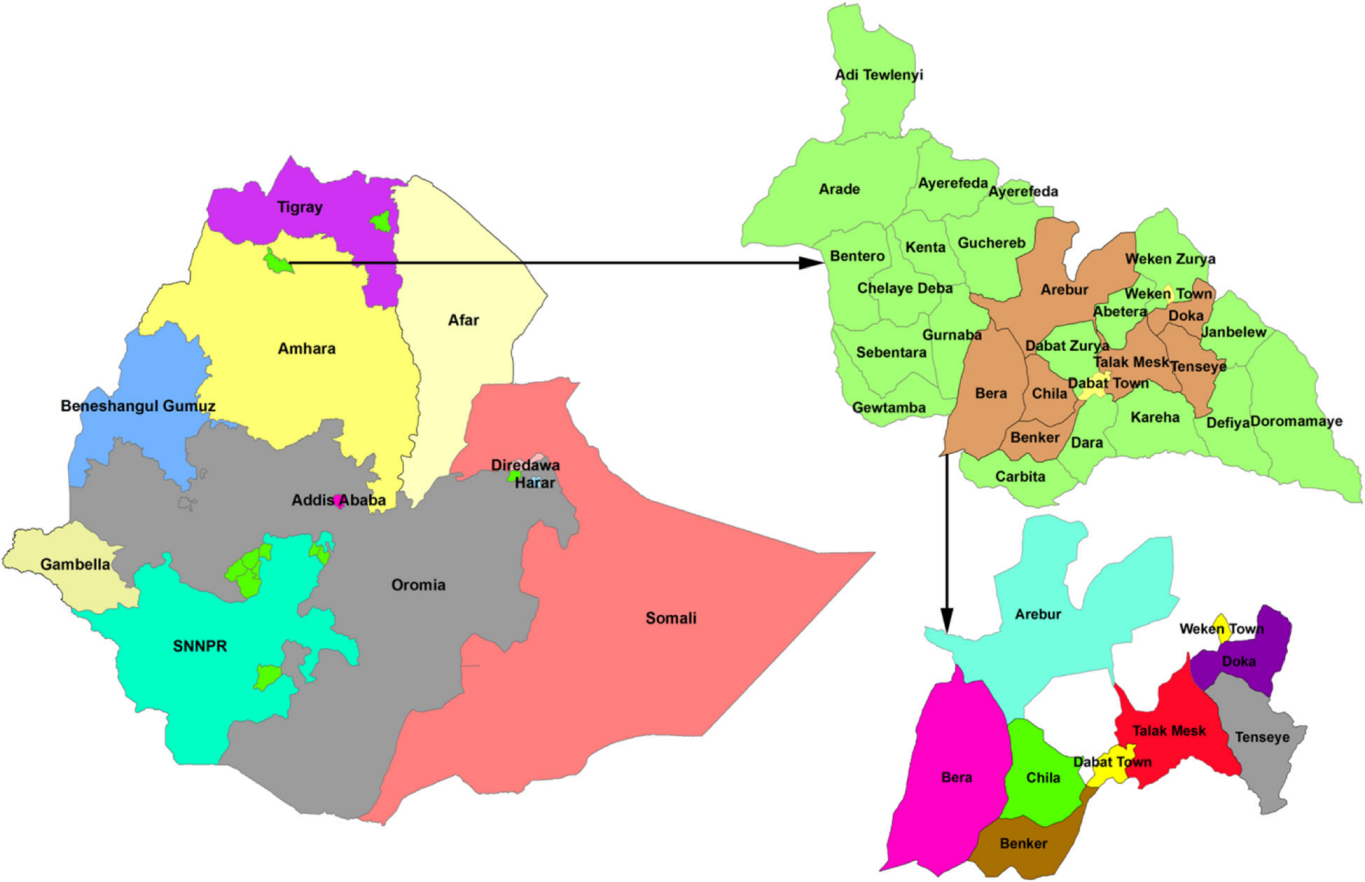
Map of the Dabat Health and Demographic Surveillance Sites (HDSS), northwest Ethiopia

The Dabat HDSS study site is located in a rural part of the Amhara regional state in northwest Ethiopia. It is 850 km northwest of Addis Ababa, the capital city of Ethiopia. The altitude of the district ranges from about 1000 to 2500 and above meters above sea level. The population consists mainly of subsistence farmers (Fig. 1). Seven rural and three urban kebeles (the lowest local administration) were included in the HDSS. According to Ethiopian Central Statistics Agency (CSA), the district had an estimated population of 145,458 residing in 27 rural and 3 urban kebeles. In the district, four health centers and 30 health posts provide health care services for the community. HDSS uses longitudinal follow-up on well-defined subjects (individuals, households, and residential units) and all related demographic and health-related outcomes within a clearly defined geographic area.

### Study design

Dabat HDSS sites follow an open dynamic cohort study design within a clearly defined geographic area. The surveillance system started with a baseline census of the population. Thereafter house-to-house visits have been done every 6 months to update data for the population in terms of all pregnancy observations and outcomes, deaths, marriage, and in-and-out migration. Causes of deaths were identified using the verbal autopsy (VA) method.

### VA data collection procedure

The VA data presented have been collected since 2007. Verbal autopsy is a method used to ascertain the cause of a death based on an interview with next of kin or other caregivers. The VA questionnaire prepared by World Health Organization (WHO) and INDEPTH (International Network of field sites with continuous Demographic Evaluation of Populations and Their Health in developing countries) was used [10–12].

A paper form platform questionnaire was adapted and translated into Amharic, the national language of Ethiopia. The cause of death, or the sequence of causes that led to death, is assigned based on the data collected through a questionnaire and any other available information. Eighteen diploma-graduated and trained and resident full-time enumerators visited the household every month for events and reported all deaths to the Dabat HDSS database. VA interviews were carried out after 45 days of the date of death in respect of the local mourning time. VA was conducted using a standardized World Health Organization (WHO) questionnaire endorsed by international Network for Demographic Evaluation of Populations and Their Health (INDEPTH network) for all deaths occurring in the HDSS [13]. For this analysis, the adult questionnaire (15 years of age and above) was used. Physicians were trained on the application of the ICD-10 manual developed by WHO for assessing the global burden of diseases [14]. The completed VA questionnaires were given to two blinded physicians and reviewed independently. When disagreements in diagnosis arose, a third physician was assigned to review the case. The final diagnosis was assigned based on agreement between the third physician and any of the two physicians. The case was considered “undetermined” if all three physicians assigned a different diagnosis. Physicians label the death as “unspecified causes of death (VA-99)” when it is difficult to classify based on the given information.

### Data quality management

The data collectors received training on questionnaires, recording, contacting close relatives, and data collection procedures. The training curriculum included sessions about individual symptoms and description in local language for easy recognition by the respondents. The VA interviewer was informed about new deaths by the resident enumerators and conduct verbal autopsy interviews. Five trained supervisors were involved in data collection and did regular supervision by rechecking the data collected by enumerators. The research team also did regular supervision to recheck the progress of data collection and sort out problems that may have been encountered by enumerators. A total of nine trained physicians (three pediatricians, three internists, and three gynecologists) were involved in assigning the causes of deaths. International Classification of Diseases (ICD-10) was used to classify main, underlying, and contributing causes of death following independent assessment of the completed questionnaires by two or three physicians [15].

### Data management and analysis

VA data entered using visual basic and exported to Stata version 12 for analysis. Frequencies, proportions, and summary statistics were used to describe the socio-demographic characteristics of the deceased individuals. Differences in causes of death among groups were reported using Pearson’s chi-square (Χ^2^) test. Binary logistic regression model was used to identify the relationship between the outcome variables (communicable diseases, non-communicable diseases, and external causes) and the independent variables. Variables in the bivariable analysis with a *P*-value of <0.2 were entered into the multivariable logistic regression analysis to control confounders. Adjusted odds ratio (AOR) with corresponding 95% confidence interval (CI) were estimated to show the strength of association. The model fitness was checked using the Hosmer and Lemeshow goodness of fit test. All statistical tests were two-sided and considered statistically significant at a *p*-value of 0.05.

## Results

### Socio-demographic characteristics of adult deaths

A total of 1082 adult deaths occurred between 2007 and 2013. Of these, 564 (52.1%) of the deaths were among females. The median age of death was 60.1 (IQR = 38.2, 73.4). The majority of the deaths (687 [63.5%]) were at ages greater than 50 years old. About 784 (72.5%) and 801 (74%) of the deceased were uneducated and self-employed, respectively. About 899 (83%) of the deaths occurred at home and 726 (67.0%) of deaths happened in mid- to lowland rural areas (Table 1).

**Table 1:** Socio-demographic characteristics of adult deaths in Dabat Demographic and Health Surveillance Site, northwest Ethiopia, 2007–2013

Characteristics	Category	Number (%)
Sex	Female	564 (52.1)
	Male	518 (47.9)
Age group (years)	Median = 60.1; IQR^a^ (38.2, 73.4)	
	15–24	122 (11.3)
	25–49	273 (25.2)
	> = 50	687(63.5)
Marital status	single	115 (10.6)
	married	546 (50.5)
	widowed	289 (26.7)
	divorced	116 (10.7)
	separated	10 (0.9)
	cohabitated	4 (0.4)
	not known	2 (0.2)
Educational status	uneducated	784 (72.5)
	1–4	138 (12.8)
	5–8	72 (6.7)
	9–12	71 (6.6)
	12+	17 (1.6)
Occupational status	Self-employed	801 (74.0)
	student	41 (3.8)
	public servant	41 (3.8)
	unemployed	16 (1.5)
	other	183 (16.9)
Place of death	Home	899 (83.1)
	Health institutions	100 (9.1)
	Others	83 (7.7)
Climatic zone	Mid- to lowland	726 (67.1)
	Highland	356 (32.9)
Year of death	2007–2010	557 (51.5)
	2011–2013	525 (48.5)

^a^ *IQR* Interquartile range

### Broad causes of death by year

**Fig. 2:**
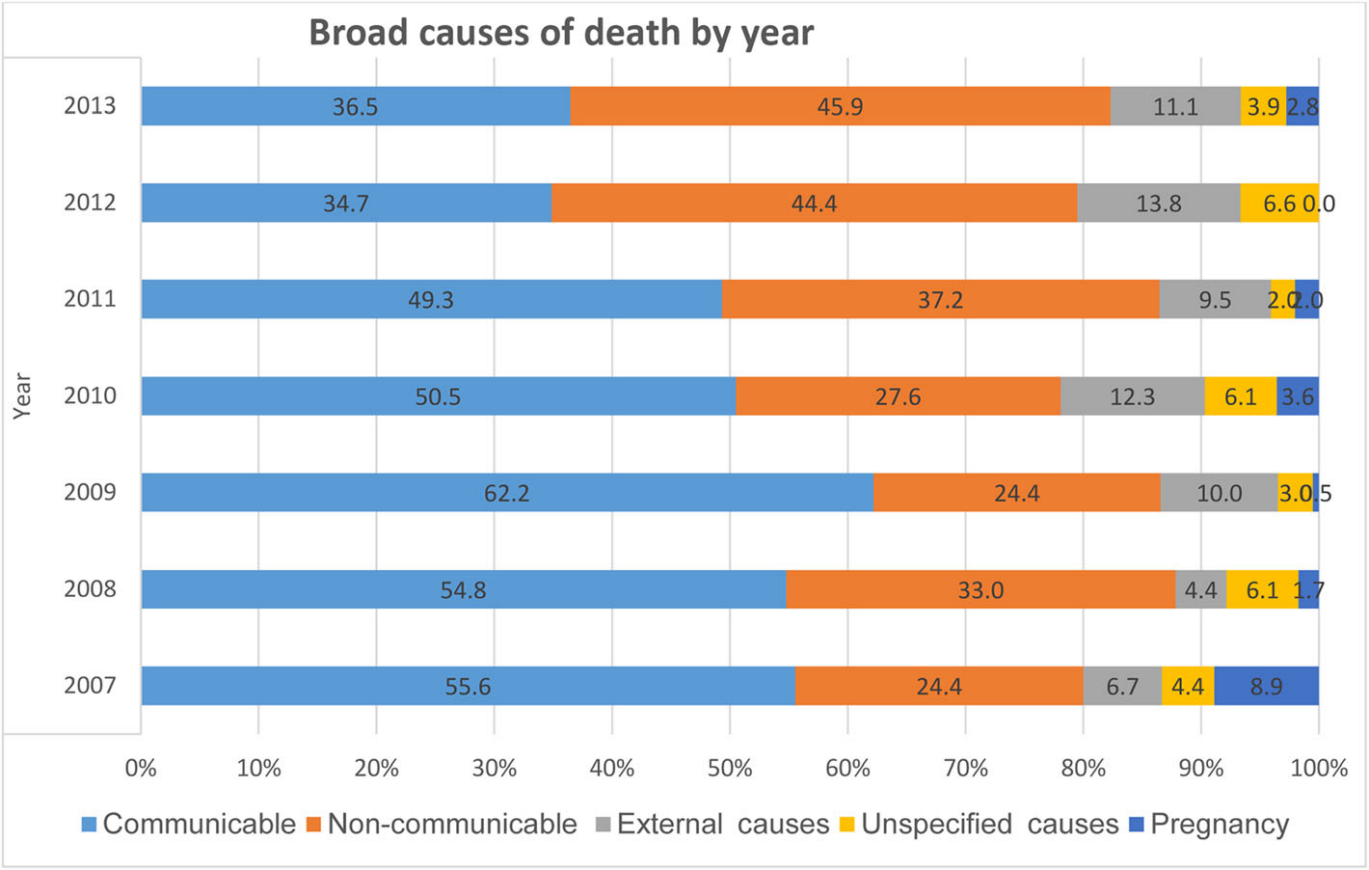
Broad causes of adult deaths by year in Dabat Demographic and Health Surveillance Site, northwest Ethiopia, 2007–2013

The highest number of deaths were registered in the years 2009 (201 deaths), 2010 (196 deaths), and 2012 (196 deaths). Of the total deaths during the period between 2007 and 2013, communicable diseases (48.0%); non-communicable diseases (NCDs) (34.8%); and external causes (10.4%) were the main causes of death in the study area. In this study, the proportion of total deaths caused by NCDs increased from 2007 to 2013. Though deaths from NCDs increased, deaths varied from year to year during the study period. Pregnancy-related causes of death were high in the year 2007 (Fig. 2).

### Specific causes of death by age

Of 1082 deaths, 687 (63.5%) were in adults age 50+, and the next highest number, 273 (25.2%), were among ages 25–49 years old. Tuberculosis (139 [20.2%]), cerebrovascular diseases (55 [8.0%]), meningitis (40 [5.8%]) and HIV/AIDS (35 [5.1%]) were the leading specific causes of death for adults 50 years old and older. Tuberculosis (54 [19.8%]), HIV/AIDS (52 [19.0%]), meningitis (19 [7.0%]), and cerebrovascular diseases (3 [1.1%]) were the leading specific causes of death for those 25–49 years old. Meningitis (17 [13.9%]) and tuberculosis (14 [11.5%]) were the leading specific causes of deaths for those 15–24 years old. Women aged 50+ were killed by tuberculosis (76 deaths [36.7%]), meningitis (30 [50.8%]), and cerebrovascular diseases (14 [27.5%]). More men were killed by malaria/leishmaniasis (11 deaths [40.7%]), and accidental falls (11 [41.7%]) compared with counterparts (Table 2).

**Table 2:** Specific causes of death by age, Dabat Demographic and Health Surveillance Site, northwest Ethiopia, 2007–2013

Specific causes of death	Female			Male			Total
	15–24	25–49	50+	15–24	25–49	50+	
	Number (%)	Number (%)	Number (%)	Number (%)	Number (%)	Number (%)	No (%)
Tuberculosis	7 (11.7)	36 (25.5)	76 (21.1)	7 (11.7)	18 (13.6)	63 (19.3)	207(19.1)
HIV/AIDS	8 (13.3)	34 (24.1)	17 (4.7)	1 (1.7)	18 (13.6)	18 (5.5)	96(8.9)
Meningitis	1 (1.7)	0	30 (8.3)	0	3 (2.3)	25 (7.7)	59(5.5)
Cerebrovascular diseases	4 (6.7)	5 (3.5)	14 (3.9)	8 (13.3)	8 (6.1)	12 (3.7)	51(4.7)
Unspecified causes of death	2 (3.3)	4 (2.8)	20 (5.5)	1 (1.7)	5 (3.8)	18 (5.5)	50(4.6)
Renal failure	2 (3.3)	2 (1.4)	19 (5.3)	1 (1.7)	5 (3.8)	17 (5.2)	46(4.3)
Congestive heart failure	4 (6.7)	3 (2.1)	24 (6.6)	1 (1.7)	3 (2.3)	10 (3.1)	45(4.2)
Acute lower respiratory infection	2 (3.3)	3 (2.1)	18 (5.0)	0	3 (2.3)	17 (5.2)	43(4.0)
Intestinal infectious diseases	1 (1.7)	2 (1.4)	14 (3.9)	2 (3.3)	1 (1.5)	13 (4.0)	33(3.0)
Malaria/leishmaniasis	1 (1.7)	3 (2.1)	7 (1.9)	2 (3.3)	3 (2.3)	11 (3.4)	27(2.5)
Hypertensive disease	4 (6.7)	4 (2.8)	9 (2.5)	1 (1.7)	2 (1.5)	5 (1.5)	25(2.3)
Intentional self-harm	0	0	12 (3.3)	0	1 (0.8)	9 (2.8)	22(2.0)
Assault	4 (6.7)	1 (0.7)	2 (0.6)	2 (3.3)	6 (4.5)	6 (1.8)	21(1.9)
Paralytic ileus and intestinal obstruction	0	1 (0.7)	1 (0.3)	2 (3.3)	9 (6.8)	4 (1.2)	17(1.6)
Accidental fall	1 (1.7)	0	3 (0.8)	2 (3.3)	2 (2.3)	9 (2.8)	17(1.6)
Acute abdomen	0	0	1 (0.3)	3 (5.0)	5 (3.8)	6 (1.8)	15(1.4)
Gastric and duodenal ulcers	0	1 (0.7)	6 (1.7)	0	3 (2.3)	5(1.5)	15(1.4)
Chronic liver disease	1 (1.7)	2 (1.4)	5 (1.4)	2 (3.3)	1 (0.8)	3 (1.5)	14(1.3)
Infectious diseases, unspecified	0	2 (1.4)	4 (1.1)	0	2(1.5)	5 (1.5)	13(1.2)
Accidental drowning and submersion	0	0	4 (1.1)	0	3(2.3)	5 (1.5)	12(1.1)
Asthma	0	0	0	6 (10.0)	3(2.3)	2 (0.6)	11(1.0)
Other causes of death^a^	20 (32.3)	36 (25.5)	75 (20.8)	19 (31.7)	27 (20.5)	55 (16.9)	232(21.4)
Total	62 (5.7)	141 (13.0)	361 (33.4)	60 (5.5)	132 (12.2)	326 (30.1)	1082

^a^Other causes of death: Malignant neoplasm of esophagus; diabetes mellitus; ischemic heart disease; contact with venomous animals and plants; malignant neoplasm of breast; other specified event, undetermined intention; pedestrian injured in traffic accident; postpartum hemorrhage; war deaths; accident, unspecified

### Specific causes of death by year

**Fig. 3:**
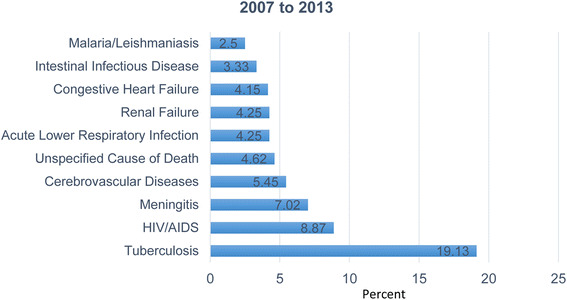
Specific causes of adult deaths by year, Dabat Demographic and Health Surveillance Site, northwest Ethiopia, 2007–2013

The majority of specific causes of death identified between 2007 and 2013 were tuberculosis (155 [20.8%]), HIV/AIDS (55 [7.4%]), and meningitis (51[6.9%]). These diseases were the top three specific causes of death from 2007 to 2011. In 2012, tuberculosis, cerebrovascular diseases, and congestive heart failure were the major specific causes of death. In 2013, tuberculosis, meningitis and cerebrovascular diseases were the major specific causes of deaths. Of the total deaths during the period of the study, 3.6% of deaths among females were due to meningitis, as were 3.3% of deaths among males. Of the total deaths, 122 (11.3%) were due to meningitis between 2007 and 2013. Of these, 76 (67.9%) occurred between 2011 and 2013, and 46 (41.0%) occurred between 2007 and 2010. The largest number of meningitis deaths (33 [29.5%]) were in the 65 and above age groups, followed by the age group between 15 and 34 (24 [21.4%]). Of the deaths due to meningitis, 67.1% occurred in rural residents (Fig. 3).

### Major specific causes of death in residence by year

The majority of specific causes of deaths among rural residents between 2007 and 2013 were tuberculosis (155 [20.8%]), HIV/AIDS (55 [7.4%]), and meningitis (51 [6.9%]). These diseases, tuberculosis (24.2%), HIV/AIDS (9.3%), and meningitis (7.7%) were the top three specific causes of death in rural areas from 2007 to 2010. In urban areas, tuberculosis (52 [15.4%]), HIV/AIDS (41 [12.2%]), cerebrovascular diseases (26 [7.7%]), and meningitis (25 [7.4%]) were the major specific causes of death between 2007 and 2013 (Table 3).

**Table 3:** Major specific causes of death by residence areas and year, Dabat Demographic and Health Surveillance Site, northwest Ethiopia, 2007–2013

Specific causes	2007–2010		2011–2013		2007–2013
	Urban	Rural	Urban	Rural	
	Number (%)	Number (%)	Number (%)	Number (%)	Number (%)
Tuberculosis	37 (19.0)	88 (24.1)	15 (10.5)	67 (17.7)	207 (19.1)
HIV/AIDS	27 (13.8)	34 (9.3)	14 (9.8)	21 (5.5)	96 (8.9)
Meningitis	18 (9.2)	28 (7.7)	7 (4.9)	23 (6.1)	76 (7.0)
Cerebrovascular disease	13 (6.7)	8 (2.1)	13 (9.1)	25 (6.6)	59 (5.5)
Unspecified causes of death	11 (5.6)	16 (4.4)	10 (7.0)	13 (3.4)	50 (4.6)
Renal failure	9 (4.6)	15 (4.1)	7 (4.9)	15 (4.0)	46 (4.3)
Congestive heart failure	10 (5.1)	8 (2.2)	10 (7.0)	17 (4.5)	45 (4.2)
Acute lower respiratory infections	6 (3.1)	16 (4.4)	4 (2.8)	17 (4.5)	43 (4.0)
Intestinal infectious diseases	5 (2.6)	18 (4.9)	1 (0.7)	9 (2.4)	33 (3.0)
Malaria/leishmaniasis	5 (2.5)	14 (3.8)	1 (0.7)	7 (1.8)	27 (2.5)
Hypertensive diseases	6 (3.1)	5 (1.4)	6 (4.2)	5 (1.3)	22 (2.0)
Intentional self-harm	0	7 (1.9)	1 (0.7)	13 (3.4)	21 (1.9)
Assault	1 (0.5)	7 (1.9)	1 (0.7)	8 (2.1)	17 (1.6)
Paralytic ileus and intestinal obstruction	1 (0.5)	13 (3.6)	1 (0.7)	2 (0.5)	17 (1.6)
Accidental fall	2 (1.0)	7 (1.9)	0	6 (1.6)	15 (1.4)
Acute abdomen	2 (1.0)	6 (1.6)	2 (1.4)	5 (1.3)	15 (1.4)
Gastric and duodenal ulcer	1 (0.5)	4 (1.1)	3 (2.1)	6 (1.6)	14 (1.3)
Chronic liver disease	3 (1.5)	2 (0.5)	2 (1.4)	6 (1.6)	13 (1.2)
Infectious diseases, unspecified	0	6 (1.6)	1 (0.7)	5 (1.3)	12 (1.1)
Accidental drowning and submersion	1 (0.5)	6 (1.6)	0	4 (1.1)	11 (1.0)
Asthma	5 (2.6)	1 (0.3)	3 (2.1)	2 (0.5)	11 (1.0)
Other specified disorders of the nervous system	1 (0.5)	4 (1.1)	2 (1.4)	4 (1.1)	11 (1.0)
Other causes of death^a^	29 (14.9)	51 (14.0)	40 (28.0)	101 (26.6)	216 (20.0)
Total	195 (18.0)	365 (33.7)	143 (13.2)	379 (35.0)	1082 (100.0)

^a^Other causes of death: Malignant neoplasm of esophagus; diabetes mellitus; ischemic heart disease; contact with venomous animals and plants; malignant neoplasm of breast; other specified event, undetermined intention; pedestrian injured in traffic accident; postpartum hemorrhage; war deaths; accident, unspecified

### Major specific causes of death by climatic zones and year

Tuberculosis (34 [15.9%]), intestinal infectious diseases (including diarrheal diseases) (15 [7.0%]), HIV/AIDS (13 [6.1%]), malaria (11 [5.1%]), and meningitis (11 [5.1%]) made up the majority of specific causes of death among lowlands and midlands dwellers. In the highlands, the majority of specific causes of death during the study period were tuberculosis (173 [19.9%]), HIV/AIDS (83 [9.6%]), meningitis (65 [7.5%]), cerebrovascular disease (53 [6.1%]), and congestive heart failure (42 [4.8%]). Of the total deceased, 776 (67.1%) lived in highlands. Of these, 388 (46.6%) were women (Table 4).

**Table 4:** Major specific causes of death by climate zones and year, Dabat Demographic and Health Surveillance Site, northwest Ethiopia, 2007–2013

Specific cause of death	2007–2010			2011–2013			2007–2013
	Highland	Midland	Lowland	Highland	Midland	Lowland	
	Number (%)	Number (%)	Number (%)	Number (%)	Number (%)	Number (%)	Number (%)
Tuberculosis	83 (21.1)	17 (29.3)	25 (23.6)	46 (13.8)	21 (29)	15 (13)	207 (19.1)
HIV/AIDS	46 (11.7)	8 (13.8)	7 (6.6)	26 (7.8)	3 (4.1)	6 (5.0)	96 (8.9)
Cerebrovascular disease	17 (4.3)	2 (3.4)	2 (1.9)	30 (9.0)	3 (4.1)	5 (4.2%)	59 (5.5)
Meningitis	23 (5.9)	3 (5.2)	4 (3.8)	13 (3.9)	2 (2.7)	6 (5.0)	51 (4.7)
Unspecified causes of death	21 (5.3)	2 (3.4)	4 (3.8)	17 (5.1)	2 (2.7)	4 (3.4)	50 (4.6)
Renal failure	18 (4.6)	2 (3.4)	4 (3.8)	14 (4.2)	4 (5.5)	4 (3.4)	46 (4.3)
Congestive heart failure	15 (3.8)	3 (5.2)	0	19 (5.7)	5 (6.8)	3 (2.5)	45 (4.2)
Acute lower respiratory infections	16 (4.1)	1 (1.7)	5 (4.7)	13 (3.9)	3 (4.1)	5 (4.2)	43 (4.0)
Intestinal infectious diseases	12 (3.1)	4 (6.9)	7 (6.6)	3 (0.9)	1 (1.4)	6 (5.0)	33 (3.0)
Malaria/leishmaniasis	12 (3.1)	0	7 (6.6)	1 (0.3)	1 (1.4)	6 (5.0)	27 (2.5)
Meningitis	12(3.1)	3 (5.2)	1 (0.9)	5 (1.5)	3 (4.1)	1(0.8)	25 (2.3)
Hypertensive diseases	9 (2.3)	2 (3.4)	0	9 (2.7)	2 (2.7)	0	22 (2.0)
Intentional self-harm	5 (1.3)	1 (1.7)	1 (0.9)	7 (2.1)	4 (5.5)	3 (2.5)	21 (1.9)
Assault	3 (0.8)	0	5 (4.7)	5 (1.5)	0	4 (3.4)	17 (1.6)
Intestinal obstruction	5 (1.3)	2 (3.4)	7 (6.6)	1 (0.3)	1 (6.7)	1 (0.8)	17 (1.6)
Accidental fall	7 (1.8)	0	2 (1.9)	2 (0.6)	0	4 (5.8)	15 (1.4)
Acute abdomen	6 (1.5)	2 (3.4)	0	5 (1.5)	1 (6.7)	1 (0.8)	15 (1.4)
Gastric and duodenal ulcer	4 (1.0)	0	1 (0.9)	7 (2.1)	1 (6.7)	1 (0.8)	14 (1.3)
Chronic liver disease	5 (1.3)	0	0	5 (1.5)	0	3 (2.5)	13 (1.2)
Infectious diseases, unspecified	3 (0.8)	0	3 (2.8)	2 (0.6)	1 (6.7)	3 (2.5)	12 (1.1)
Accidental drowning and submersion	5 (1.5)	0	2 (1.9)	1(0.3)	0	3 (2.5)	11 (1.0)
Asthma	5 (1.5)	1 (1.7%))	0	4(1.2)	0	1 (0.8)	11 (1.0)
Other specified disorders of the nervous system	4 (1.0)	1 (1.7%)	0	5 (1.5)	0	1 (0.8)	11 (1.0)
Malignant neoplasm of esophagus	0	0	0	7 (2.1)	0	3 (2.5)	10 (0.9)
Diabetes mellitus	3 (0.8)	0	0	4 (1.2)	0	2 (1.7)	9 (0.8)
Ischemic heart disease	3 (0.8)	0	1 (0.9)	5 (1.5)	0	0	9 (0.8)
others causes of death^a^	51 (13.0)	4 (6.9)	18 (17.0)	77 (23.1)	15 (20.5)	28 (23.5)	193 (17.8)
Total	393 (36)	58 (5.3)	106 (9.8)	333 (30.8)	73 (6.7)	119 (11)	1082 (100)

^a^Other causes of death**:** Contact with venomous animals and plants; malignant neoplasm of breast; other specified events, undetermined intention; pedestrian injured in traffic accident; postpartum hemorrhage; war deaths; accident, unspecified; other specified neoplasms; puerperal sepsis

### Major specific causes of death among women ages 15–49 and above

HIV/AIDS (8 [12.9%]), meningitis (8 [12.9%]), tuberculosis (7 [11.3%]), and other maternal causes (6 [9.6%]) were the major specific causes of death among women aged 15–49 years. Tuberculosis (139 [20.2%]) and cerebrovascular diseases (55 [8.0%]) were the most common specific causes of death for women aged 50 and older.

### Factors associated with specific causes of death

The variables sex, residence, age group, and education status were significantly associated after adjusting for possible confounding variables. Females were at 1.4 (95% CI: 1.1–1. 8) times greater risk of death due to communicable diseases compared with their male counterparts. The odds of death from communicable disease are 1.5 times higher among rural residents than urban (95% CI: 1.1–2.1). Age groups between 25 and 49 years are the highest-risk age group for communicable diseases. The risk of dying from communicable diseases for the age group 25–49 years was 1.6 times higher than in the 15–24 age group (95% CI:1.0–2.5). Those who completed high school or greater were at 3.4 times higher risk of death from communicable diseases compared with those who were not educated (95% CI: 1.1–10.1) (Table 5).

**Table 5:** Factors associated with communicable diseases, non-communicable diseases and external causes of adult deaths in northern Ethiopia, 2007–2013

Covariate	Level	No Number (%)	Yes Number (%)	Crude odds ratio (95% CI)	Adjusted odds ratio (95% CI)
Communicable diseases					
Sex	Female	276 (49.0)	288 (55.4)	1.3 (1.0, 1.6)	1.4 (1.1, 1. 8)
	Male	287 (51.0)	231 (44.6)	1	1
Residence	Urban	189 (33.6)	148 (28.5)	1	1
	Rural	374 (66.4)	371 (71.5)	1.3 (1.0 1.6)	1.5 (1.1; 2.1)
Age group	15–24 years	71 (12.6)	51 (9.8)	1	1
	25–49 years	128 (22.7)	145 (27.9)	1.6 (1.0; 2.4)	1.6 (1.0; 2.5)
	> = 50 years	364 (64.7)	323 (62.2)	1.2 (0.8; 1.8)	1.3 (0.9; 2.0)
Education	Uneducated	405 (71.9)	379 (73.0)	1	1
	1–4	76 (13.5)	62 (11.9)	0.9 (0.6; 1.3)	1.0 (0.7;1.5)
	5–8	42 (7.5)	30 (5.8)	0.8 (0.5: 1.2)	0.9 (0.5;1.6)
	9–12	35(6.2)	36 (6.9)	1.1 (0.7; 1.8)	1.6 (0.85;3.02)
	12+	5 (0.9)	12 (2.3)	2.6 (0.9; 7.4)	3.4 (1.1;10.1)
Climatic zone	Mid-lowland	111 (19.7)	103 (19.8)	1	
	Highland	452 (80.3)	416 (80.2)	1.0 (0.7; 1.3)	1.1 (0.8;1.5)
Non-communicable diseases					
Sex	Female	361 (51.2)	176 (54.0)	1	1
	Male	344 (49.8)	150 (46.0)	0.9 (0.7; 1.2)	0.9 (0.7; 1.2)
Residence	Urban	192 (27.2)	128 (39.3)	1.7 (1.3, 2.3)	1.7 (1.2, 2.4)
	Rural	513 (72.8)	198 (59.7)	1	1
Age group	15–24 years	91 (12.9)	28 (8.6)	1	
	25–49 years	209 (29.6)	56 (17.2)	0.9 (0.5;1.5)	0.8 (0.5; 1.3)
	> = 50 years	405 (57.4)	242 (74.2)	1.9 (1.2;3.1)	1.8 (1.1; 3.0)
Education	Uneducated	501 (71.1)	239 (73.3)	1	
	1–4	94 (13.3)	41 (12.6)	0.8 (0.6; 1.4)	1.0 (0.6;1.5)
	5–8	45 (6.4)	26 (8.0)	1.2 (0.7; 2.0)	1.5 (0.9; 2.7)
	9–12	53 (7.5)	15 (4.6)	0.6 (0.3; 1.1)	0.6 (0.3;1.2)
	12+	12 (1.7)	5 (1.5)	0.9 (0.3; 2.5)	0.7 (0.2; 2.2)
Climatic zone	Mid- and lowland	158 (22.4)	47 (14.4)	1	1
	Highland	547 (71.6)	279 (85.6)	1.7 (1.2;2.5)	1.4 (0.9; 2.0)
External causes					
Sex	Female	540(55.7)	24 (21.2)	1	
	Male	429(54.3)	89 (78.8)	4.7 (2.9; 7.5)	4.5(2.8; 7.4)
Residence	Urban	316(32.6)	21 (18.6)	1	
	Rural	653(67.4)	92 (81.4)	2.1 (1.3; 3.5)	1.7 (0.9; 3.2)
Age group	15–24 years	93 (9.6)	29 (25.6)	4.7 (2.8, 7.8)	3.9 (2.2, 7.1)
	25–49 years	232 (23.9)	41 (36.3)	2.6 (1.7, 4.2)	2.6 (1.7, 4.3)
	> = 50 years	644 (66.5)	43 (38.1)	1	**1**
Education	Uneducated	720 (74.3)	64 (56.6)	1	
	1–4	114 (11.8)	24 (21.2)	2.4 (1.4; 3.9)	1.3 (0.7;2.2)
	5–8	59 (6.1)	13 (11.5)	2.5 (1.3; 4.8)	1.5 (0.7; 3.1)
	9+	76 (7.8)	12 (10.6)	1.8 (.9; 3.4)	1.3 (0.6; 3.0)
Climatic zone	Mid- and lowland	175 (18.1)	39 (34.5)	1	1
	Highland	794 (81.9)	74 (65.5)	0.4 (0.3; 0.7)	0.5 (0.3; 0.9**)**

Place of residence and age have significant association with non-communicable diseases as causes of death. The odds of death due to non-communicable diseases were 1.5 times higher in rural residents than urban 1.5 (1.1–2.1). Older ages (50 and above) have a 1.8 (95% CI:1.1–3.0) times higher risk of death due to non-communicable diseases as compared to the 15–24 age group. However, the association with climatic zone was not statistically significant after adjusting for confounding. External causes of death were associated with sex, age group, and climatic zone. Males had a 4.5 times higher risk of death from external causes than females (95% CI: 2.8–7.4). The chances of death due to external causes were high in the 15–24 age group (3.9 [2.2–7.1]) and the 25–49 age group (2.6 [1.7–4.3]) compared to the 50+ group (Table 5).

Of all specific causes of death, tuberculosis, HIV/AIDS, and meningitis were found to be the top three causes of death in the study area. The covariates residence and climatic zone were significantly associated with tuberculosis. Of all those deceased due to tuberculosis, most were rural residents and lived in the highland climatic zone. The risk of death from tuberculosis was 1.6 (95% CI: 1.10–2.4) times higher among rural residents than urban. With respect to climatic zone, highlanders had a 1.5 (95% CI: 1.0–2.3) times higher risk of death due to tuberculosis compared to other causes (Table 6). Sex, age, and education were significantly associated with HIV/AIDS as the cause of death in Dabat district. Females had a 1.8 (95% CI: 1.2–3.0) times higher risk of death due to HIV/AIDS than males. Deaths at ages 25–49 years were 3.2 (95% CI: 1.5–6.9) times more likely to be due to HIV/AIDS. Education status was also associated with causes of death: those who completing high school were 3.1 (95% CI: 1.5–6.5) times more likely to die of HIV/AIDS than those who were uneducated. For meningitis as a possible cause of death, only age was significantly associated. Age groups 25–49 years and 50 + years were at 0.5 (95% CI: 0.2–0.9) and 0.4 (95% CI: 0.2–0.7) times lower risk of death, respectively, due to meningitis as compared to the 15–24 year age group (Table 6).

**Table 6:** Factors associated with the top three causes of death, Dabat Demographic and Health Surveillance Site, northwest Ethiopia, 2007–2013

Covariate	Level	No	Yes	COR (95% CI)	AOR (95% CI)
		Number (%)	Number (%)		
Tuberculosis					
Sex	Female	445 (50.9)	119 (57.5)	1	
	Male	430 (49.1)	88 (42.5)	0.8 (0.6; 1.0)	0.8 (0.7; 1.1)
Residence	Urban	285 (32.6)	52 (25.1)	1	
	Rural	590 (67.4)	155 (74.9)	1.4 (1.0; 2.0)	1.6 (1.1; 2.4)
Age group	15–24 years	108 (12.3)	14 (6.8)	1	
	25–49 years	219 (25.1)	54 (26.1)	1.9 (1.0; 3.6)	1.9 (1.0; 3.5)
	> = 50 years	548 (62.6)	139 (67.1)	1.9 (1.1; 3.5)	1.7 (0.9; 3.2)
Education	Uneducated	621 (71.0)	163 (78.7)	1	
	1–4	114 (13.0)	24 (11.6)	0.8 (0.5; 1.3)	0.9 (0.6; 1.5)
	5–8	65 (7.4)	7 (3.4)	0.4 (0.2; 0.9)	0.5 (0.2;1.2)
	9+	75 (8.6)	13 (6.3)	0.6 (0.3; 1.1)	0.8 (0.4;1.7)
Climatic zone	Mid- and lowland	180 (20.5)	34 (16.5)	1	
	Highland	696 (79.5)	172 (85.5)	1.3 (0.9; 2.0)	1.5 (1.0; 2.4)
HIV/AIDS					
Sex	Female	505 (51.2)	59 (64.5)	1.5 (1.0, 2.3)	1.8 (1.2, 3.0)
	Male	481 (49.8)	37 (38.5)	1	1
Residence	Urban	296 (30.0)	41 (42.7)	1	
	Rural	690 (70.0)	55 (57.8)	0.6 (0.4; 0.9)	1.0 (0.6; 1.7)
Age group	15–24 years	113 (11.5)	9 (9.4)	1	
	25–49 years	221 (22.4)	52 (54.2)	3.0 (1.4; 6.2)	3.2 (1.5; 6.9)
	> = 50 years	652 (66.1)	35 (36.5)	0. 7 (0.3; 1.4)	1.0 (0.4; 2.3)
Education	Uneducated	733 (74.3)	51 (53.1)		
	1–4	124 (12.6)	14 (14.6)	1.6 (0.9; 3.0)	1.7 (0.9; 3.3)
	5–8	62 (6.3)	10 (10.4)	2.32 (1.1; 4.8)	1.8 (0.8; 4.1)
	9+	67 (6.8)	21 (21.9)	4.5 (2.6; 7.9)	3.1 (1.5; 6.5)
Climatic zone	Mid- and lowland	201 (20.4)	13 (13.4)		
	Highland	786 (79.6)	83 (86.5)	1.6 (0.9; 3.0)	1.38 (0.7; 2.7)
Meningitis					
Sex	Female	524 (52.1)	40 (52.6)	1	1
	Male	482 (47.9)	36 (47.4)	1.0 (0.6; 1. 6)	1.0 (0.6; 1.8)
Residence	Urban	312 (45.0)	25 (32.9)	1	1
	Rural	694 (55.0)	51 (67.1)	0.9 (0.6;1.5)	1.0 (0.6;2.2)
Age group	15–24 years	105 (10.4)	17 (22.4)	1	1
	25–49 years	254 (25.2)	19 (25.0)	0.5 (0.2; 0.9)	0.4 (0.2; 0.9)
	> = 50 years	647 (64.3)	40 (52.6)	0.4 (0.2; 0.7)	0.4 (0.2; 0.7)
Education	Uneducated	732 (72.8)	52 (68.4)	1	1
	1–4	132 (13.1)	6 (8.0)	0.6 (0.3; 1.5)	0.5 (0.2; 1.4)
	5–8	66 (6.6)	6 (8.0)	1.9 (0.5; 3.1)	0.9 (0.4; 2.4)
	9+	76 (7.6)	12 (15.8)	2.2 (1.2; 4.4)	1.7 (0.7; 3.8)
Climatic zone	Mid- and lowland	203 (20.2)	11 (14.5)	1	1
	Highland	803 (79.8)	65 (85.5)	1.5 (0.8; 2.9)	1.7 (0.8; 3.4)

## Discussion

In this study, tuberculosis, HIV/AIDS, and meningitis were the most common specific causes of death among adults. The study identified the rise in deaths due to non-communicable diseases. The majority of deaths occurred among those aged 50 + years. Tuberculosis and cerebrovascular diseases were the leading specific causes of death for adults 50 and older. Tuberculosis and HIV/AIDS were the leading specific causes of death for those 25–49 years old. Meningitis and tuberculosis were the leading specific causes of death for those 15–24 years old. In rural dwellers, tuberculosis, HIV/AIDS, and meningitis were the main causes of death, whereas in urban areas, tuberculosis, HIV/AIDS, cerebrovascular diseases, and meningitis were the major specific causes of death among adults.

Communicable and non-communicable diseases, and external causes were the leading broad causes of deaths in the study area. The finding is consistent with the evidence from Dodowa HDSS that the major causes of death were communicable diseases followed by non-communicable disease [16]. In Kilete Awulalo HDSS, northern Ethiopia, the leading causes were non-communicable diseases followed by communicable diseases and external causes [7]. This study indicates that nearly half of the causes of death in the adult population were due to these problems. In an urban slum of Ludhiana, non-communicable diseases contributed to the majority of deaths [17].

Of all specific causes of deaths, tuberculosis, HIV/AIDS, and meningitis were found to be the top three causes of death in the study area. The study done in northern Ethiopia identified that tuberculosis, cerebrovascular diseases, and accidental falls were the leading specific causes of deaths [7]. In the present study, tuberculosis and HIV/AIDS were the dominant causes of death in the study population. Similar findings came from a study done in Butajira rural DHSS, where the most common causes of death were reported to be tuberculosis and HIV/AIDS. In Nairobi, Kenya, Urban HDSS showed the most common causes of death in the country were HIV/AIDS and tuberculosis [18]. In West Africa Abidjan, AIDS and tuberculosis were the leading causes of death in adult men. In Italy, the fourth leading cause of death among men between the ages of 25 and 44 years and among Swiss women and men aged 25 to 44 years [19–21]. In a rural population in KwaZulu-Natal, South Africa, HIV-related illness (including tuberculosis) accounted for 50% deaths [22]. The risk of death from tuberculosis was higher among rural residents than urban. Highlanders had a higher risk of death due to tuberculosis. Males had a 46% lower risk of death due to HIV/AIDS as compared to females. Meningitis was one of the top causes of death among adults and was high in rural highland dwellers. Pneumococcal meningitis is associated with high mortality and morbidity rates in adults [23]. Bacterial meningitis is a grave disease of high incidence, especially in less developed countries.

Non-communicable diseases such as cardiovascular conditions and injuries have become increasingly important contributors to deaths among adults. The similar evidence from Nairobi Urban HDSS supported this finding, [24] and in KwaZulu-Natal, South Africa, HDSS, non-communicable lifestyle-related conditions accounted for 15.0% of deaths [22]. In low- and middle-income countries, it is estimated that more than 21.0% of deaths are due to cardiovascular diseases [2]. In this study, non-communicable diseases accounted for nearly 35.0% of deaths in the adult population, and cardiovascular disease alone accounts for 5.5% of deaths in this population. Though the burden of non-communicable diseases seems much less in low- and middle-income countries, it shows an increasing trend. Cerebrovascular diseases as causes of death among ages 50 and above were high [25, 26]. Non-communicable diseases are the leading causes of death globally, killing more people each year than all competing causes combined [27].

In the present study, the proportion and trend of NCDs as causes of death increased considerably across the 5 years compared with communicable diseases. Ethiopia is facing a dual burden of increasing NCDs combined with the existing burden of infectious diseases. As a result, the country has to deal with the double burden of communicable and non-communicable diseases [28]. The effect of demographic changes, such as aging, rapid unplanned urbanization, and nutritional change contributes for burden of NCDs [29, 30]. Place of residence and age have significant association with non-communicable diseases as causes of death. The chance of death due to non-communicable diseases was 41.0% lower for rural residents than urban. The 50 and above age group has a higher risk of death due to non-communicable diseases as compared to younger age groups. This finding is in line with other studies [31].

External causes of death were associated with climatic zone, sex, and age group of the deceased. Males have a 4.5 times higher risk of death due to external causes than females. This may be because males have more frequent exposure to risk factors for external causes of death compared to females. This finding is consistent with other studies [32]. Those 50 years old and older had a 75% reduction in deaths due to external causes compared to younger age groups. This is in agreement with another study conducted in India [32]. In this study, intestinal infectious diseases were one of the major causes of death among lowlanders, where availability of safe water might be limited due to inaccessibility and other potential factors. Studies have shown that in developing countries, intestinal infections are common due to contaminated drinking water sources and contribute to causes of death [33, 34]. The decline in these causes of deaths observed in this study might be due to changes resulting from the cumulative efforts of partners and the government of the country to improve water, sanitation, and hygiene [35].

Males were 28.0% less at risk of death due to communicable diseases compared to females. This finding is consistent with the studies conducted in a different part of Ethiopia in which the odds of dying due to communicable diseases were higher among females than males [7, 36]. The chance of death from communicable disease was 50.0% higher among rural residents than urban. Age groups between 24 and 49 years are at greatest risk of death from communicable diseases. There was a 60.0% higher risk of dying due to communicable diseases for the 25–49 year age group as compared to the 15–24 year age group. These findings are similar to those of a study done in Addis Ababa [32, 36]. Deaths due to communicable diseases were three times higher for those who finishing secondary school as compared to those who were not educated, a finding which is also supported by another study [31].

VA is recognized as the only feasible alternative to comprehensive medical certification of deaths in settings with no or unreliable vital registration systems [37, 38]. However, doubts have remained about the ability of VA to provide accurate and timely information about the causes of deaths in populations. VA is time-consuming and expensive, and it is difficult to maintain the quality of cause assignment on a large scale over long periods of time [39, 40]. Irrespective of the inherent limitation of VA in leveling causes of death, the finding from the surveillance site has described the importance of VA in detecting specific causes of death in resource-limited countries where death registration is not yet available [41]. The VA questionnaire included a “free text” section for the interviewer to record an open narrative provided by the respondent about the terminal illness and events. The open text could help physicians verify causes of death. However, if the physician relies only on the open narrative to diagnose causes of death, this could affect the physician reviewers in formulating a diagnosis of the probable cause of death.

This study demonstrated the possibility of ascertaining at least the leading causes of death, reducing the misclassification of causes of death, and deriving the probable underlying cause of death when it has not been reported. This study has limitations of retrospective data collection: waiting until after the 45-day mourning period to conduct interviews raised the risk of missing information and detection bias. There is a risk of misclassification of the reason of death for HIV, because it could be due to TB and/or meningitis. The inability to trace and report signs and symptoms of a deceased individual correctly prior to death by the interviewee and challenges with physicians agreeing on the probable cause of death based on already collected data were factors in the high rate of undetermined causes of death.

## Conclusion

This study showed that the majority of the deaths among adults were from tuberculosis, HIV/AIDS, and meningitis. Deaths due to non-communicable diseases showed an increasing trend*.* Males, rural residents, and members of the 25–49 age group were at higher risk of death due to communicable diseases, whereas rural residence and older ages (50 and above) were significantly associated with deaths due to non-communicable diseases. Increasing community awareness of infections and their interrelationships, intensifying tuberculosis case finding, implementing effective local TB programs, and providing successful treatment and increased interventions for HIV are of supreme importance.

## Abbreviations

CSA: Central Statistics Agency
HDSS: Health and Demographic Surveillance System
ICD-10: International Classification of Diseases
INDEPTH: International Network for Demographic Evaluation of Populations and Their Health
NCD: Non-communicable diseases
VA: Verbal autopsy
